# Self-Efficacy as an Agentic Protective Factor against Death Anxiety in PTSD and Psychiatric Co-Morbidity

**DOI:** 10.1007/s11126-019-09694-5

**Published:** 2019-12-04

**Authors:** Mark Hoelterhoff, Man Cheung Chung

**Affiliations:** 1 School of Health in Social Science, Medical School, University of Edinburgh, Doorway 6 Teviot Place, Edinburgh, EH8 9AG UK; 2 The Chinese University of Hong Kong, Hong Kong, China

**Keywords:** Agency, Protective factors, Self-efficacy, Death anxiety, PTSD, Psychiatric Co-Morbidity

## Abstract

PTSD has profound personal, social and economic impact. Understanding factors that influence strong recovery is a priority for informing the use of limited treatment resources. This study follows up a preliminary finding from Hoelterhoff and Cheung Chung, Psychiatr Q, 88, 635-651, [30] which found that death anxiety is related to PTSD and suggested that self-efficacy may mediate this relationship. Specifically, this study examined self-efficacy as a protective factor in the context of people who have experienced a life-threatening event. 109 undergraduate university students completed self-report questionnaires on, self-efficacy, death anxiety, trauma and well-being as well as a number of demographic factors. Self-efficacy was found that to be significantly and inversely related to death anxiety and psychiatric co-morbidity, but not PTSD. Results were discussed in light of literature regarding death anxiety. It seems that self-efficacy is related to death anxiety and well-being; however, it interacts with these processes independently and not as a mediating factor. More research is needed to understand coping mechanisms that help develop resilience against the negative effects of death anxiety against PTSD and minimize its detrimental impact on mental health.

## Introduction

In a previous study done by Hoelterhoff and Cheung Chung [30], a mixed methods investigation examined how death anxiety influenced PTSD and mental health among people who have experienced a life-threatening event. Data indicated significant correlation between death anxiety and PTSD, but not psychiatric co-morbidity. In phase 2 of that study, a qualitative study attempted to further explore the phenomenological experience of participants with full PTSD; results indicated that agentic themes emerge in response to the life-threatening event. Overall these agentic coping mechanisms functioned as protective factors allowing participants to develop resilience against the effects of death anxiety and minimize its negative impact on mental health [30]. If agentic protective factors are the key to managing the detrimental effects of death anxiety, can a sense of control within a strength-based perspective be fundamental to this process?

Self-efficacy is an idea that addresses issues of control and the belief in oneself as having the strengths and resources to handle problems that may arise [4, 33, 37]. In Bandura’s social cognitive theory, perceived self-efficacy to exercise control over potential threats plays a central role in anxiety arousal. By examining the relationship perceived between self-efficacy and anxiety arousal, research shows perceived coping “inefficacy” is accompanied by high levels of subjective distress, autonomic arousal and catecholamine secretion [4]. Analyses of the causal structure of self-protective behaviour show that anxiety arousal and avoidant behaviour are mainly co-effects of perceived coping inefficacy [2, 4]. This idea is a key concept to keep in mind when examining the clinical implication of intrusive negative thinking and anxiety arousal of PTSD.

Currently, there is limited literature that could propose a useful model of therapeutic resilience (based in part on self-efficacy) which would mediate death anxiety with PTSD and psychiatric co-morbidity. Previous literature predominantly focuses on death as a specific fear or anxiety and as something that should be grieved, perhaps in the context of psychotherapy [6, 32]. However, previous research has identified components that will help develop a framework in which to understand the interaction. People’s self-efficacy, then, includes a sense of competency in relation to death; low competency results in high death anxiety and vice versa [40]. The emphasis is coping with death itself. Self-efficacy then becomes an issue of control or at least a perceived level of control over the end of one’s life. People are afraid of death despite whether or not that awareness fluctuates. Thus, death anxiety is understood as being controlled by death competency, in other words a death self-efficacy [24, 28, 40].

Previous studies have found self-efficacy to be an important proactive “agentic” factor in post-traumatic recovery. Self-efficacy has been consistently linked with well-being and improved psycho-social functioning [4, 7, 10, 31]. Specifically, in regard to anxiety, self-efficacy has been shown to help individuals exert more control over their anxious thought processes [3, 4, 19, 46, 51].The social cognitive model of death anxiety focuses on this type of perceived self-efficacy. People perceive or believe that they have the ability to prepare for death and the issues surrounding death. This issue of perceived control correlates with the anxiety around death. A review of death anxiety literature shows self-efficacy is both a predictor and mediator of death anxiety [19, 46].

People with low levels of self-efficacy are more likely to report high levels of death anxiety [11, 50]. People’s beliefs about their own self-efficacy are linked to various determinants of death anxiety. Indeed, a related review has found that people who are anxious about death also show a tendency towards hampered belief in self-ability and mental health difficulties [32]. Death anxiety is consistently found to correlate negatively with difficulties in perceptions of self [1, 16, 35, 38].

The role of self-efficacy with regard to death anxiety appears to tap into a range of skills, beliefs and attitudes about self and death. If self-efficacy serves as a protective or coping factor in death anxiety and life-threatening events, it is important to examine the relationship between self-efficacy and PTSD. At this stage it is important to propose a theoretical model in light of the previously mentioned research. Based on the social-cognitive theory of post-traumatic recovery, the agentic adaptation model can be used to demonstrate the mediational role of self-efficacy between death anxiety and PTSD/psychiatric co-morbidity. This theory has its roots in an agentic perspective that views people as self-organising, proactive, self-reflecting and self-regulating, not just as reactive organisms shaped by traumatic forces or driven by inner pathology [4]. This agentic model has been applied to PTSD symptomology. This agentic model would then assert that death anxiety is a result of the reduction of resources that relates self-efficacy thus impacting PTSD.

The proposed model suggests that a “loss of resources” results from the impact of a life-threatening event which challenges both physiological and psychological resources. Second, this loss of resources then triggers self-efficacy as a coping strategy. Thirdly, depending on the level of self-efficacy, PTSD symptoms will either be manageable or result in clinically high symptoms of hyperarousal, intrusion and avoidance [7]. Therefore, it can be theorised that the agentic model can also be applied to death anxiety in a similar fashion to PTSD. Death anxiety from a life-threatening event creates a loss of resources. As stated earlier, people fluctuate in their level of awareness of death anxiety; however, the life-threatening event brings the death anxiety to the forefront of consciousness. The death anxiety is then akin to the lack or loss of resources in the adaptation model of PTSD [18]. In the agentic process, self-efficacy would be triggered as a coping resource for the individuals. Therefore, a person’s ability to remain resilient against death anxiety in the face of a life-threatening event would be mediated by self-efficacy.

The agentic model as applied to the experience of death anxiety is a relevant and slightly modified model of the social cognitive perspective on PTSD. The reason it has been adapted for the purpose of studying death anxiety is that literature suggests death anxiety has a negative effect of psychological resources [26, 48]. According to the literature, death anxiety affects general psychological functioning; therefore, using the agentic model, self-efficacy should act as a mediator. Higher self-efficacy should mediate the effects of death anxiety resulting in lower mental health difficulties. Conversely, lower self-efficacy would be less effective in mediating the impact of death anxiety on mental health. In order to develop this model of death anxiety resilience further, research needs to demonstrate that death anxiety is mediated by self-efficacy and thus has demonstrable effects on PTSD and mental health outcomes.

Previous studies have shown self-efficacy and factors relevant to self-efficacy are significantly related to death anxiety [1, 11, 16, 19, 32, 35, 38, 50, 46]. The main conclusion from this research is that higher self-efficacy should act as an effective mediator of death anxiety on mental health, thus the rationale for the proposed agentic model of death anxiety resilience.

## Research Aims

The aim of this study is to explore the relationship between death anxiety, self-efficacy, PTSD and psychiatric co-morbidity. Thus, this study will examine three hypotheses

1. Death anxiety is significantly related to self-efficacy
2. Self-efficacy is significantly related to PTSD
3. Self-efficacy is significantly related to psychiatric co-morbidity

**Fig. 1:**
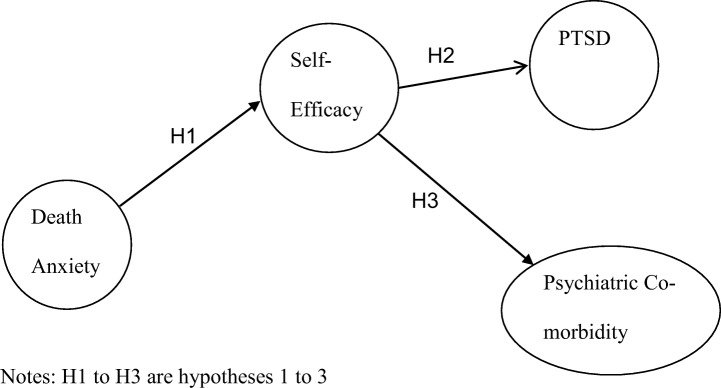
Hypothesis Model H1-H3

The following model will be tested. See Fig. 1.

## Method

### Participants

A population of 109 students at a Lithuanian university participated in the study (M = 26, F = 83) with an average age of 20.63 (SD = 1.86). Almost all (93.6%) were single. Most (66.1%) were Lithuanians and the rest were predominantly from Northern and Eastern Europe. Over half (62.4%) were an upper level year group (years 2 and 4) at the time of the study.

### Procedure

Prior to recruitment, significant time was taken to create both Lithuanian and Russian versions of the questionnaires. This was carefully done by developing two translation teams each comprised of 4 members of the university translation club. They were supervised directly by the first author. Upon creation of the questionnaires, they were reviewed by member of the university linguistics department for accuracy. The questionnaires were then back translated into English and compared to the original assessments to improve the accuracy of representing the original English questionnaires. The questionnaires were available in English, Lithuanian and Russian. Although English is the language of instruction, the researcher intended that participants could choose to take the questionnaires in the language in which they were most comfortable.

Prior to recruitment, the study was submitted to the university International Review Board for approval and was given clearance to be conducted after all ethical and procedural issues were considered. Participants were recruited during a social science module lecture with the tutor’s permission. To secure credits for that module, students engaged in different academic activities, one of which was to participate in a research project. Students also had the option not to engage in any of these activities.

This researcher gave out the self-administered questionnaires during the lecture and was available to participants to clarify unfamiliar terms. Student volunteers collected the surveys. This researcher then explained to the class the theory being tested as an additional supplement to the module material.

Questionnaires consisted of:

1. Participant demographics
2. Posttraumatic Stress Diagnostic Scale (PDS)
3. General Health Questionnaire-28 (GHQ-28)
4. Death Anxiety Scale (DAS)
5. The General Perceived Self-efficacy Scale (PSSS)

Participants who indicated at the beginning of the demographic questionnaire that they had never experienced a life-threatening event were assigned to the control group. If participants answered “no” to having a traumatic event, they were asked to bypass the PDS. They were still asked to complete the assessments on the PSSS, DAS and the GHQ-28.

### Measures

1. Demographic information:

- Age
- Gender
- Year level
- Marital status
- National identity
- Homesickness
- Stress from independent living
- Comfortable with living arrangements
- First time living without parents
- Coursework deadline within the next 7 days
  2) The Posttraumatic Stress Diagnostic Scale (PDS) [18] assesses symptoms resulting from experiencing a traumatic event, according to DSM-IV criteria. Participants were classified into the following groups: ‘no PTSD’ (not meeting the diagnostic criteria) and ‘PTSD’ (meeting all required symptom criteria for re-experiencing, avoidance and hyperarousal along with helplessness, terror and an impact upon daily function). In part I, information was gathered about the trauma itself. Participants are asked to identify whether they have experienced any past life-threatening events including natural disasters, accidents, physical or sexual assault, combat or exposure to a war zone, sudden violent or unexpected death of someone close, and life-threatening injury. Questions were asked on trauma exposure characteristics (if yes); number of events, most severe trauma, how long ago trauma happened, physically injured, someone else injured, was life in danger, someone else’s life in danger, feel helpless, feel terrified?

Part II assesses PTSD symptoms resulting from having experienced the most life-threatening experience. It is composed of the 17 questions contained in the DSM-IV diagnostic criteria and generates three subscales: re-experiencing, avoidance and hyperarousal. Participants were asked to rate the severity of symptoms: 0 = not at all, 1 = once a week or less/once in a while, 2 = 2 to 4 times a week/half the time, 3 = 5 or more times a week/almost always. Excellent internal consistency (alpha = 0.97), and test-retest reliability (0.96) have been recorded over a 2 to 3 day period. The PDS is known to be strongly correlated with other measures of PTSD such as the Mississippi Scale (0.93) and the Impact of Event Scale (0.90). This scale has shown good reliability, validity and good agreement with the Structured Clinical Interview for Diagnosis (kappa = 0.65, agreement = 82%, sensitivity = 0.89 and specificity = 0.75).

3) The General Health Questionnaire-28 (GHQ-28) [25] measures general psychological morbidity and global dysfunction, and yields four subscales: somatic problems, anxiety, social dysfunction and depression. The GHQ-28 has shown a sensitivity value of 88% at a specificity of 84.2% and an overall misclassification rate of 14.5%.
4) The Death Anxiety Scale *(DAS)* is self-report instrument for measuring death anxiety which consists of 15 true or false items. Templer’s 15 item scale, first published in 1970, has been used extensively and has been translated into many different languages. It has been shown to have test-retest reliability of 0.83, and an internal consistency coefficient of 0.73 (Templar, 1970).
5) Perceived Self-Efficacy Scale

The scale was created to assess perceived self-efficacy with the aim to predict coping with daily problems, as well as adaptation after experiencing all kinds of stressors [42]. It is self-administered and scoring responses are made on a 4-point scale and then summed. The responses to all 10 items yield the final composite score with a range from 10 to 40. The construct of Perceived Self-Efficacy reflects an optimistic self-belief; beliefs that one can perform difficult tasks, and cope with adversity in various domains of human functioning. Perceived self-efficacy taps internal-stable attributions and facilitates goal-setting, effort investment, persistence in face of barriers and recovery. Perceived self-efficacy is an operative construct and is related to subsequent behaviour and, therefore, is relevant for clinical treatment [42]. The measure has been used internationally with success for two decades and in samples from 23 nations [42]. The scale is uni-dimensional and criterion-related validity is documented in numerous correlation studies where positive coefficients were found with favourable emotions, dispositional optimism, and work satisfaction [42]. Negative coefficients were found with depression, anxiety, stress, burnout, and health complaints [42].Therefore it can be taken to predict adaptation after traumatic life changes, but it is also suitable as an indicator of quality of life at any point in time. The Self-Efficacy scale was analysed using item alpha reliability and showed good internal consistency (α = 0.843).

### Data Analysis Plan

Descriptive statistics were used to describe the demographic information of the participants. T-test, Chi-Square and multivariate analysis variance were used to compare the life-threatening event and control groups in terms of the differences of mean and percentage scores. Correlation coefficients including point biserial correlation (r_pb_) were used to establish the relationship between demographic variables and outcome variables. Point biserial correlation was used when one of the variables in the correlational analysis was dichotomous. Partial Least Squares (PLS) analysis was used to examine the interrelationship between the constructs in the hypothesized model. The mediation procedure recommended by Baron and Kenny [5] and the Sobel test were used to investigate mediational relationships identified in the final model.

The assumptions and diagnostics related to multiple linear analysis were examined. Three subscales of the GHQ-28 (somatic, anxiety, social dysfunction) were subjected to a logarithmic transformation. When performing regression analysis to examine mediational relationships, one outlier was detected during the exploration of diagnostics (Mahalanobis ≥3 SD) and was subsequently removed from the analysis. Following exploration and transformation, assumptions relating to multivariate normality, linearity and homoscedasticity were met. Regression imputation was used in order to address the missing data. In this study, less than 1% of the missing data was imputed which was deemed to be acceptable in literature [41].

## Results

Fifty-two (47.7%) participants reported life-threatening events and 57 (52.3%) did not. Almost a fifth (17.6%) experienced one event while almost half (49%) at least two events. Remaining participants experienced three or more events. According to the PDS, 24 people (22%) met diagnostic criteria for full PTSD and 28 (25.6%) did not. Table 1 shows the demographic information of the PTSD, no-PTSD and control groups.

**Table 1:** Demographic details

	PTSD group		No PTSD group		Control	
	Mean	SD	Mean	SD	Mean	SD
Age	20.58	1.74	20.35	2.07	20.78	1.75
	N	%	N	%	N	%
Male Female	2 22	8.3 91.7	10 18	35.7 64.3	14 43	24.6 75.4
Level						
Year 1 Year 2 Year 3 Year 4	4 3 8 9	16.7 12.5 33.3 37.5	9 3 3 13	32.1 10.7 10.7 46.4	17 5 12 23	29.8 8.8 21.8 40.4
Marital Status						
Single Married Co-habituating	23 1	95.8 4.2	24 2 2	85.7 7.1 7.1	55 1 1	96.5 1.8 1.8
What country is passport from?						
Lithuania Latvia Belarus Russia Ukraine USA Other	14 3 2 1 2 2	58.3 12.5 8.3 4.2 8.3 8.4	23 2 2 1	82.1 7.1 7.1 3.6	35 2 6 5 2	61.4 12.3 3.5 10.5 8.8 3.6
	Mean	SD	Mean	SD	Mean	SD
Homesickness	3.56	1.71	2.94	0.96	3.40	1.79
Stress from independent living	2.57	1.80	1.91	1.01	2.13	1.37
Comfort with living arrangements	5.21	1.27	6.20	0.76	5.39	1.26
	N	%	N	%	N	%
First time without parent						
No Yes	12 12	50 50	20 8	71.4 28.6	42 15	73.7 26.3
Deadline in 7 days						
No Yes	17 7	70.8 29.2	22 6	78.6 21.4	51 6	89.5 10.5

There were no significant differences between groups participating in the study when considering age [F(2,73) = 1.75, ns], gender (Fisher’s exact test χ^2^ = 5.44, df = 2, ns), year level (χ^2^ = 1.08, df = 2, ns), marital status (Fisher’s exact test χ^2^ = 3.36, df = 2, ns), nationality, (χ^2^ = 4.42, df = 2, ns), degree of homesickness [F(2,73) = 0.82,ns], stress from independent living [F(2,73) = 1.75, ns], how comfortable they were with living arrangements [F(2,73 = 2.45, ns], first time living without parents (χ^2^ = 4.56, df = 2, ns), or having a coursework deadline in 7 days (Fisher’s exact test χ^2^ = 0.21, df = 2, ns).

Table 2 shows the event which was most bothersome in the group of participants who experienced a life-threatening event. Comparing the life-threatening groups, there were no significant differences between the full PTSD and no-PTSD group regarding the number of events (t = 1.48, df = 49, ns). In addition, both the full PTSD group and no-PTSD group on average experienced the life-threatening event which bothered them the most just over four years ago (t = 0.26, df = 50, ns). The frequencies of identified experiencing specific life-threatening events between groups were quite similar, see Table 2.

**Table 2:** Life-threatening event which bothered the most

	PTSD Group		No PTSD group	
Life threatening events	Number	%	Number	%
Serious accident	6	25.0	8	28.6
Physical assault by family member or someone you know	5	20.8	5	17.8
Physical assault by a stranger	5	20.8	9	32.1
Sexual assault	1	4.2	0	0
Life-threatening illness	7	29.2	6	21.5
	Mean	SD	Mean	SD
Onset of the event (in months)	55.37	52.16	51.50	54.12
Number of life-threatening events	2.73	1.35	2.21	1.16

Table 3 summarises the means and standard deviations of psychiatric co-morbidity, death anxiety, and self-efficacy. GHQ has a clinical cut-off at 4; DAS has clinical case cut-off. Results showed significant differences between the three groups for anxiety [F (2,105) = 3.42, *p* < 0.05]. Post Hoc (LSD) analysis showed that the full PTSD group had higher levels of anxiety than the no- PTSD group (p < 0.05). There was also a significant difference between the three groups for social dysfunction [F (2,105) = 6.14, *p* < 0.01], full PTSD higher in severity than no-PTSD (p < 0.01) and control (p < 0.01) (Post Hoc LSD). In addition, there was a significant difference in depression [F (2, 105) = 6.44, p < 0.01], full PTSD was higher in severity than no-PTSD (p < 0.01) and control (p < 0.01) (Post Hoc LSD). However, there were no significant differences for somatic problems [F (2, 105) = 3.00, ns]. There was no significant difference between PTSD, no-PTSD or control on death anxiety [F (2,105) = 3.03, ns] or self-efficacy [F (2,105) = 1.75, ns].

**Table 3:** Mean and standard deviations for DAS, SE, and GHQ-28

	PTSD Group		No PTSD Group		Control	
	Mean	STD	Mean	STD	Mean	STD
GHQ-28						
Somatic	17.16	4.39	14.78	3.24	15.43	3.65
Anxiety	16.79	5.38	13.46	3.28	15.61	4.94
Social Dysfunction	17.33	2.89	14.85	2.12	15.57	2.66
Depression	13.41	5.31	9.60	2.91	10.56	3.64
DAS						
Total	8.70	2.88	6.96	2.78	7.61	2.28
Self-efficacy						
Total	30.68	5.23	33.13	3.11	30.80	3.98

Prior to the PLS analysis of establishing the relationship between death anxiety, self-efficacy, PTSD and psychiatric co-morbidity, the demographic variables needed to be controlled. To this end, correlation coefficients including point biserial correlations (r_bp_) were carried out to see which demographic variables were related to outcome. The results show that gender was the only significant variable associated with PTSD, possibly due the difference in number for of men and women in the sample. Unfortunately, due to the fact that PLS analysis requires multiple indicators for each construct, the current research could not develop the construct of demographic information with gender being the only indicator. Ergo, gender was not included in the PLS analysis, however PTSD scores were compared between males and females; results showed female participants reported more PTSD symptoms than males (female mean = 4.08, sd = 1.59 vs. Male m = 2.38, sd = 1.59, t = −2.98, df = 45, *p* < 0.01).

To test the hypothesized model of the relationships between death anxiety, self-efficacy, PTSD and psychiatric co-morbidity, we carried out partial least squares (PLS) analysis using PLS-Graph 3.00 [13, 14]. PLS is an alternative to standard structural equation modelling (SEM). PLS models, like SEM models, incorporate latent variables (constructs) with multiple indicators. One of the advantages of PLS over SEM, and a major factor in choosing it for the current work, is that, in contrast to SEM which requires a large sample size, it can be used with modest sample sizes even for relatively complex models. Arguably, the sample size in this study was too small for SEM.

Unlike SEM, PLS makes no distributional assumptions and models may incorporate formative, as well as reflective, indicators. Having multiple indicators of the construct would increase the reliability of what the construct represents. PLS generates outer and inner model estimates. The outer model estimates refer to the loadings or weights for each indicator and show how strongly it relates to the construct. The inner model estimates refer to the linear relationship between constructs by means of regression coefficients. PLS does not generate a test of model fit but provides estimates of path coefficients for the paths in the model and tests of whether these path coefficients differ significantly from zero. The tests were carried out using bootstrap re-sampling to generate t statistics. Two hundred bootstrap samples were produced.

Less than 1% of responses were missing due to participants omitting questionnaire items. PLS have no procedures for dealing with incomplete observations so regression imputation was used to replace the missing data. Regression imputation has been shown to be a valid method in dealing with missing data [41]. It should be noted that PLS analysis requires multiple indicators for each construct. Therefore, in situations where there are not specific subscales, multiple indicators needed to be created. In order to provide multiple indicators for death anxiety and self-efficacy, 3 item and 2 item parcels were computed respectively. Death anxiety indicators were created by using questions that reflected three types; fear of dying, death caused by external circumstances and thinking about death. The indicators of self-efficacy were created by taking the items from the scale and dividing them into two items: SE1, SE2.

**Fig. 2:**
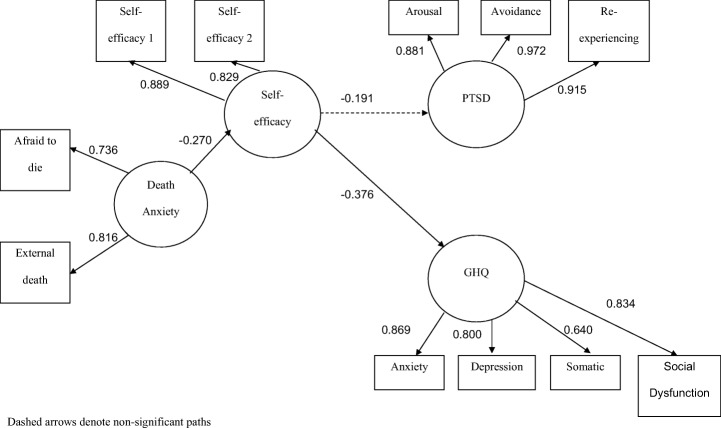
PLS Pathway analysis

Table 4 shows the estimated loadings of the scale items death anxiety (afraid to die, thoughts of death, & external death), self-efficacy (self-efficacy 1 and self-efficacy 2), PTSD (Intrusion, avoidance & hyperarousal) and psychiatric co-morbidity (somatic problems, anxiety, social dysfunction & depression). Reflective indicators with loadings that were not significantly different from zero were removed to ensure construct validity. Accordingly, the indicator of thinking about death was dropped from the PLS analysis. The correlation matrix for the indicators used in the modelling is given in Table 5. The final structural model can be seen in Fig. 2. The resulting path coefficients for relationships between constructs are shown in the figure which also indicates their significance. Unlike SEM, PLS analysis does not tell us the degree of model-fit. Instead, it examines predictive capability of the model characterized by the presence of strong construct loadings (>0.60), standardized path coefficients (>0.20) and at least moderate R^2^ values. Most of the construct loadings were strong. The path coefficients of the significant paths were also strong (see later) and the average R^2^ of the overall model was 0.0626 (average communality = 0.5306, average redundancy = 0.0381). Death anxiety was significantly associated with self-efficacy (B = -0.2700, SE = 0.1571, *p* < 0.05) which was in turn associated with psychiatric co-morbidity (B = -0.3760, SE = 0.1299, p < 0.05).

**Table 4:** Loadings and weights of Indicators on the constructs (latent variables)

Latent variable	Indicator^*a*^	SE	Loading
Death anxiety	Afraid to die	0.3633	0.7199*
	Thoughts of death	0.3477	0.5543†
	External death	0.3829	0.8183*
Self-efficacy	Self-efficacy 1	0.1235	0.8835**
	Self-efficacy2	0.2582	0.8354*
PTSD	Intrusion	0.1491	0.9149**
	Avoidance	0.1401	0.9718**
	Hyperarousal	0.0992	0.8812**
Psychiatric co-morbidity	Somatic problems	0.2155	0.6385*
	Anxiety	0.0937	0.8683**
	Social dysfunction	0.1207	0.8343**
	Depression	0.1283	0.8008**

^a^All indicators are reflective indicators

*p < 0.05; **p < 0.001; Significance levels are based on bootstrapped standard errors

† Dropped items

**Table 5:** Estimated correlations between the transformed variables used in the PLS model

	Afraid Die	Thought Death	Ext Death	Soma	Anx	SocDys	Dep	Intru	Avoid	Arou	SE1	SE2
Afraid Die	1.0	0.246	0.208	0.087	0.055	0.296*	0.195	0.206	0.185	0.036	−0.253	−0.330*
Thought Death		1.0	0.442**	−0.020	0.293*	0.129	0.183	0.169	0.366*	0.250	−0.052	−0.147
Ext Death			1.0	0.119	0.210	0.103	0.126	0.043	0.198	0.208	−0.336*	−0.122
Soma				1.0	0.605**	0.442**	0.290*	0.343*	0.205	0.419**	−0.282	0.031
Anx					1.0	0.581**	0.562**	0.364*	0.305*	0.427**	−0.472**	−0.180
SocDys						1.0	0.569**	0.260	0.263	0.292*	−0.311	−0.417*
Dep							1.0	0.456**	0.474**	0.346*	−0.378	−0.405*
Intru								1.0	0.755**	0.755*	−0.083	−0.001
Avoid									1.0	0.709**	−0.282	−0.221
Arou										1.0	−0.024	−0.030
SE1											1.0	0.585**
SE2												1.0

AfraidDie = feeling that you were going to die, ThoughtDeath = having thoughts about death, ExtDeath = fear something would kill you,

Soma = somatic problems, Anx = anxiety, Social = social dysfunction, Dep = depression, Intru = intrusion, Avoid = avoidance behaviour, Arou = hyperarousal, SE1 = Self efficacy pt. 1, SE2 = Self-efficacy pt. 2

*p < 0.05, **p<

Conceptually, path results suggest that participants’ self-efficacy mediated the relationship between death anxiety and psychiatric co-morbidity. To verify this, we examined mediation using the regression procedure recommended by Baron and Kenny [5]. To begin with, significant association between the independent variable (IV) and dependent variable (DV) need to be established. Then, for complete mediation, three conditions need to be met: a) The IV must be significantly associated with the mediator, b) the mediator must be significantly associated with the DV, c) the relationship between the IV and DV becomes non-significant when the mediator is controlled. For partial mediation, the three conditions remain the same except that for condition c, the relationship between the IV and the DV remains significant when the mediator is controlled.

Results showed that death anxiety (IV) was not correlated with psychiatric co-morbidity (DV) (B = 0.075, SE B = 0.039, Beta = 0.265, ns) suggesting that the first condition of the mediation analysis was not met. Given this, the rest of the mediation procedure did not need to be carried out. In other words, self-efficacy was not a mediator between death anxiety and psychiatric co-morbidity. This lack of mediation was confirmed using the Sobel test. The results showed that self-efficacy did not mediate the relationship between death and psychiatric co-morbidity (Z = 0.93, ns).

## Discussion

The aim of this study was to explore the relationship between death anxiety, self-efficacy, PTSD and psychiatric co-morbidity. Further, specifically to look at an agentic model of death anxiety resilience in the context of PTSD. The results highlight that self-efficacy is related to death anxiety supporting the first hypothesis of this study. This result is consistent with previous research and demonstrates a connection between self-efficacy and death anxiety [1, 11, 16, 19, 20, 21, 32, 35, 38, 50, 46].

Surprisingly, self-efficacy was not found to be related to PTSD and the second hypothesis was rejected. This specific finding is inconsistent with previous research which identifies self-efficacy as related to posttraumatic symptoms [4, 7, 39, 49]. However, some studies have indicated that other factors in combination with self-efficacy demonstrate greater influences on PTSD [8, 12, 15]. The third hypothesis was supported, however, and self-efficacy influences psychiatric co-morbidity. Other research has shown self-efficacy is correlated positively with mental health [4, 7, 27].

One strength of this study is that the sample population experienced a range of life-threatening events, whereas many studies have examined very specific types of trauma. Despite other studies’ focus on specific populations and subsequent findings, this study has yielded similar results. This indicates that the findings can be seen as somewhat robust in that it is not event or setting specific. While on one hand this may be considered a strength, it may conversely be seen as a weakness. The results of study show self-efficacy is related to psychiatric co-morbidity, but not PTSD. This is surprising, considering the previously mentioned literature review on self-efficacy and PSTD. Research shows the association between self-efficacy and PTSD is transient. Self-efficacy is associated with short term adjustment to traumatic events; however, this association tends to diminish over time [23, 43]. Yet, other studies suggest that self-efficacy and trauma may not be closely associated. For example, an increase in self-efficacy was not followed by improvement in PTSD symptoms [17, 47].

However, if there are ample studies showing a likely link between self-efficacy and PTSD as previously mentioned, it begs the question as to why the relationship was not evidenced in this study. Going back to trauma types, this may have influenced the results. This is not a study on a specific type of traumatic event, e.g., sexual abuse, PTSD and self-efficacy. Hence the broad nature of trauma types and life-threatening events in this sample may have reduced the strength of the relationship. Additionally, the age of the sample may have influenced the lack of connection of self-efficacy to PTSD. Interestingly, research has been inconsistent on the effects of age on PTSD; some research has shown the severity of PTSD increases with age, while others suggests context for trauma is more significant [36, 44].

The connection to psychiatric co-morbidity indicates a strong relationship to self-efficacy and may be symptom specific, but not necessarily specific to PTSD symptoms. In other words, self-efficacy, in actuality, relates more to symptoms of general psychological difficulties & global dysfunction. It can be argued that self-efficacy is more relevant to general psychological difficulties or global dysfunction versus being specific to PTSD. For example, it was found that self-efficacy related to the immediate mental health difficulties from trauma, but not the long-term symptomology of PTSD [9]. How is this plausible? As stated earlier, self-efficacy relates to general anxiety arousal on the whole. Perhaps what should be considered is that self-efficacy regulates a more general sense of health anxiety as stated in the cognitive behavioural literature ([20], 1997). This is also possible because PTSD and psychiatric co-morbidity could be considered as two different syndromes. In any case, in this sample self-efficacy is related to psychiatric co-morbidity.

Self-efficacy did affect psychiatric co-morbidity, although not in the way proposed by this researcher. Death anxiety relates to self-efficacy; however, self-efficacy did not mediate the impact of death anxiety on psychiatric co-morbidity. This was surprising because previous studies suggest that death anxiety is positively related to mental health. Ergo, the degree to which mental health problems are exacerbated by death anxiety should be mediated by self-efficacy. Perhaps what can be said is that although death anxiety directly influences PTSD, self-efficacy improves general mental health. By decreasing mental health difficulties, in essence, the relationship of death anxiety to PTSD may be indirectly influenced via self-efficacy. Literature shows that when death anxiety is controllable via self-efficacy and the person has less psychiatric co-morbidity, it improves the perceived level of manageability regarding PTSD [39]. Conversely, if people have uncontrollable death anxiety due to low self-efficacy and high psychiatric co-morbidity, they will have a more difficult time managing PTSD.

However, in regard to the earlier stated model of death anxiety resilience, it should be noted that self-efficacy was not demonstrated to be a mediator; it does not carry the influence of death anxiety to psychiatric co-morbidity which contradicts the agentic model of death anxiety. Self-efficacy relates to death anxiety independently of self-efficacy relating to psychiatric co-morbidity implying that these are separate psychological processes. It was proposed that self-efficacy is a mediator in the agentic model, but the results do not support this. In light of the results that self-efficacy contributes to death anxiety and contributes to psychiatric co-morbidity, it should be considered that this is an additive, not mediational, model of death anxiety. This is not inconsistent with Bandura’s model of self-efficacy because, in its essence, it is an intrinsic human response to cope with death anxiety and it is an intrinsic human response that also copes with psychiatric co-morbidity [20]. If indeed death anxiety and psychiatric co-morbidity are two different psychological phenomena, even though self-efficacy is related to both, they are different processes. One way to consider the role of self-efficacy is as a “surge protector” that buffers against ordinary psychological reactions so they do not become maladaptive. The results show a negative relationship of self-efficacy to death anxiety and psychiatric co-morbidity. Self-efficacy seems to protect against “normal” emotional affect from becoming maladaptive, without this protection it could develop into psychopathology.

Returning to the subject of PTSD, perhaps it does not demonstrate the same effect because it results from an extraordinary event. According to the results of this study, self-efficacy does not necessarily buffer against the distress related to PTSD. However, the agentic model is not fully contradicted by this study. Perhaps death anxiety and loss of resources are quite different. As stated earlier, this study is based on young adults, not necessarily representing the population used in Bandura’s research regarding PTSD. In his model, self-efficacy is something that develops across the lifespan and is related to cognitive development [3]. In other words, the use of self-efficacy specifically with those suffering from PTSD may not be as developed with a younger population.

It’s important to consider academic stressors influencing the sample population. However, the results do not support this idea; none of the added demographic information (Homesickness, Stress from independent living, Comfort with living arrangements, first time living without parents, Coursework deadline within the next seven days) demonstrated significance. Considering limitations of this study, there may be other issues of distress that have not been accounted for that are affecting this sample. These may be factors that exist within the general population. Data from the World Health Organization rank Lithuania as one of the worst standardized death rates from suicide and self-inflicted injury [29]. This very high rate of suicide is complex and difficult to determine in its causality; however, it does lend itself to speculation on overall mental health difficulties. Mortality from mental disorders (including alcohol dependency) was the highest in Europe in 1995 [29]. The registered prevalence of mental diseases among the total population was 4.2% in 1998 and epidemiological studies show that almost 500,000 people may need psychological, psychotherapeutic or psychiatric counselling [29]. Obviously, these are generalisations about the people of that region, but should be considered as potential influences on the levels of psychiatric co-morbidity in the sample.

Considering some of the limitations of this study, it is important to note that this is not a longitudinal study; therefore, causality cannot be determined from the results. Although it would be beneficial for future research, in this case it was not practical. Due the specifics of the setting and the short-term placement of this researcher, longitudinal follow-up would have been very difficult and labour intensive. Regardless of the benefits of a longitudinal design, the other themes of religious coping and existential attitude needed to be explored. Unfortunately, it would not be feasible to do a longitudinal study of all three themes. In addition, due to this being a cross-sectional design, it cannot be established as to whether this is a state or trait phenomenon. This is indeed a limitation in a majority of death anxiety research [34, 45]. A cross-sectional study does not allow for this type of discussion. Finally, PLS analysis does not establish causality; it works with regression and associations between variables. In other words, it is correlational and directional, but not causal. In light of this, the results must be interpreted in light of the aforementioned design limitations.

One final consideration is the cross-cultural implications for self-efficacy. This researcher’s anecdotal experience of the culture led to an investigation as to whether self-efficacy is actually more of a western concept. Indeed, literature does show self-efficacy is more congruent with a western focus on self-reliance, achievement and mastery [22]. These values may not be as socially desirable in the northern and eastern European context. Future research should explore whether self-efficacy is indeed a universal coping mechanism relevant cross-culturally, specifically for death anxiety and mental health.

## Conclusion

This study has demonstrated the role of self-efficacy as influencing both death anxiety and psychiatric co-morbidity, separately. However, the role of self-efficacy between death anxiety and PTSD is complicated. The social cognitive perspective for understanding death anxiety may still be useful. It appears as though self-efficacy switches on for death anxiety and psychiatric co-morbidity, but not for PTSD. Psychologists and mental health workers should acknowledge the clinical reality of death anxiety among victims of trauma. It is indeed linked to well-being and should be considered within a psychotherapeutic treatment plan. Psychologists and mental health workers should both recognise and encourage victims of trauma to utilise coping mechanisms. These may include self-efficacy as a way to buffer against death anxiety and promote resilience to it. The results do indicate a relationship there and further research could explore interventions that support people who’ve suffered traumatic events by bolstering self-efficacy.
